# To the Editors of the Medical and Physical Journal

**Published:** 1801-05

**Authors:** E. Bevan

**Affiliations:** Stoke-upon-Trent


### *To the Editors of the Medical and Phyſical Journal.*

Gentlemen,

I Think it a duty at this period, to diffuſe as widely as poſſible every circumſtance which can tend in any degree to throw light on ſo important a ſubject as the Vaccine Inoculation, and thereby contribute either to remove the prejudices or confirm the opinions of thoſe who oppoſe its introduction. I am induced by this conſideration to ſend you the following account, and requeſt that you will give it a place in your Journal.

On Monday, the 12th ult. I was ſent for to the wife of Robert Baker, near Newcaſtle-under-Lyne, whom I found affected with the Small-pox, and in the ſeventh month of her pregnancy: The eruption, which was very copious and univerſal, firſt appeared upon the preceding Thurſday, the day on which I ſaw her being therefore the fifth day of its appearance. As ſhe had two children who had not had the Small-pox, I conſidered it as a fair opportunity of putting to the teſt the preventive power of the Cow-pock, and immediately inoculated them with Vaccine matter. As the Small-pox frequently proves fatal to pregnant females, it may not be amiſs to obſerve, that in Mrs. Baker's caſe, (though ſhe was very much loaded) there were no alarming ſymptoms, and that ſhe is now completely recovered. The inflammation and ſuppuration went on in the inoculated parts with perfect regularity, attended with ſcarcely any perceptible indiſpoſition, and one of the children complained of conſiderable tenderneſs in the axilla. They both lived in the ſame apartment with their mother during the whole progreſs of her illneſs, and on the 23d and 24th, (the 12th and 13th days after inoculation) I was ſomewhat alarmed for the credit of the Vaccine Inoculation, (on which I placed the fulleſt reliance) by the appearance of ſymptoms in both children, reſembling thoſe which take place at the approach of Small-pox, viz. ſickneſs, thirſt, head-ache, laſſitude, &c. On the 25th theſe ſymptoms totally diſappeared, but recurred upon the 28th, on which day and the following, about ſixty eruptions appeared upon one child, and about twenty on the other, which eruptions exactly reſembled thoſe of Small-pox in every reſpect, excepting that they arrived at maturity on the 30th, being the third day of their appearance. From the time of their firſt appearance the children have been free from complaint.

From the above facts, it appears probable, that the Vaccine Inoculation had produced a change in the ſyſtem, though not ſuch as completely to prevent the influence of the variolous contagion, contagion,* yet ſufficient to mitigate the violence of its effects, and indeed to occaſion the diſeaſe to deviate from its natural progreſs. I am, &c.

*Stoke-upon-Trent, Feb.* 11, 1801. E. BEVAN.

---

### *To the Editors of the Medical and Phyſical Journal.*

* We think that this eruption was not variolous. ED.

